# Bioactive Secondary Metabolites from *Phomopsis* sp., an Endophytic Fungus from *Senna spectabilis*

**DOI:** 10.3390/molecules19056597

**Published:** 2014-05-22

**Authors:** Vanessa Mara Chapla, Maria Luiza Zeraik, Valdecir F. Ximenes, Lisinéia Maria Zanardi, Márcia N. Lopes, Alberto José Cavalheiro, Dulce Helena S. Silva, Maria Cláudia M. Young, Luiz Marcos da Fonseca, Vanderlan S. Bolzani, Angela Regina Araújo

**Affiliations:** 1 NuBBE - Núcleo de Bioensaios, Biossíntese e Ecofisiologia de Produtos Naturais, Departamento de Química Orgânica, Instituto de Química, UNESP, Universidade Estadual Paulista, Araraquara-SP, 14800-900, Brazil; E-Mails: vanessachapla@gmail.com (V.M.C.); marialuizaze@gmail.com (M.L.Z.); lisineia@hotmail.com (L.M.Z.); mnlopes@iq.unesp.br (M.N.L.); albjcava@iq.unesp.br (A.J.C.); dhsilva@iq.unesp.br (D.H.S.S.); bolzaniv@iq.unesp.br (V.S.B.); 2 Departamento de Química, Faculdade de Ciências, UNESP, Universidade Estadual Paulista, Bauru-SP, 17033-360, Brazil; E-Mail: vfximenes@fc.unesp.br; 3 Instituto de Botânica, Núcleo de Pesquisa em Fisiologia e Bioquímica, São Paulo-SP, 04301-902, Brazil; E-Mail: marxyoungmc@gmail.com; 4 Departamento de Análises Clínicas, Faculdade de Ciências Farmacêuticas de Araraquara, UNESP, Universidade Estadual Paulista, Araraquara-SP, 14801-902, Brazil; E-Mail: fonseclm@fcfar.unesp.br

**Keywords:** secondary metabolites, bioactivities, endophytic fungi, *Phomopsis* sp., *Senna spectabilis*

## Abstract

Chemical investigation of an acetonitrile fraction from the endophytic fungus *Phomopsis* sp. led to the isolation of the new natural product 2-hydroxy-alternariol (**7**) together with the known compounds cytochalasins J (**1**) and H (**2**), 5'-epialtenuene (**3**) and the mycotoxins alternariol monomethyl ether (AME, **4**), alternariol (AOH, **5**) and cytosporone C (**6**). The structure of the new compound was elucidated by using 1-D and 2-D NMR (nuclear magnetic resonance) and high resolution mass spectrometry. The cytochalasins J (**1**) and H (**2**) and AOH (**5**) exhibited potent inhibition of the total ROS (reactive oxygen species) produced by stimulated human neutrophils and acted as potent potential anti-inflammatory agents. Moreover, cytochalasin H (**2**) demonstrated antifungal and acetylcholinesterase enzyme (AChE) inhibition *in vitro*.

## 1. Introduction

Endophytes are microorganisms that inhabit plant interiors, especially the leaves, stems and roots, with no apparent harm to their host [1,2]. These microorganisms have received considerable attention over the last 20 years since their ability to protect a host against insects and pathogens was noted [3]. This protection is associated with biologically active compounds isolated from the endophytes. Special attention has been given to the presence of mycotoxins in grains that carry endophytes primarily because some of these secondary metabolites, such as alternariol (AOH) and alternariol monomethyl ether (AME), are toxic to both humans and animals [4] and are responsible for spoilage in grains, fruits and vegetables [5]. The presence of mycotoxins in the natural environment and in foodstuffs has been reported as an agricultural problem for several decades [4].

Brazilian Cerrado trees are well-known sources of bioactive secondary metabolites [6,7,8,9], of which *Senna spectabilis* (Fabaceae) was chosen for a detailed microbiological investigation. This plant presented several endophytes, including *Phomopsis* sp., which were chemically and biologically investigated. 

**Figure 1:**
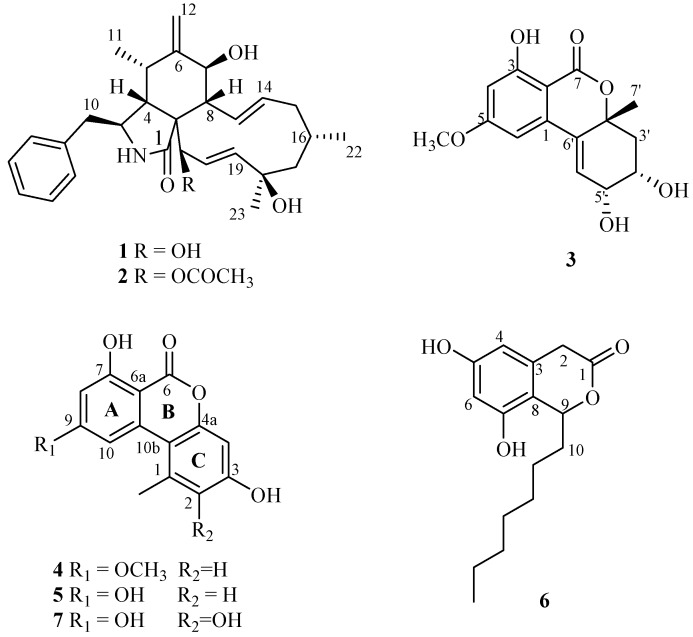
Chemical structures of **1**–**7** produced by *Phomopsis* sp.

We report the isolation of cytochalasin J (**1**) and H (**2**), 5'-epialtenuene (**3**), alternariol monomethyl ether (**4**), alternariol (**5**), cytosporone C (**6**) and the new natural product 2-hydroxyalternariol (**7**) (Figure 1). Furthermore, the antioxidant, anti-inflammatory, antifungal and cytotoxic activities of these compounds were evaluated.

## 2. Results and Discussion

Column chromatography and preparative HPLC were used to isolate the six known compounds: cytochalasin J (**1**), cytochalasin H (**2**) [10,11,12], 5'-epialtenuene (**3**) [13,14], alternariol monomethyl ether (AME) (**4**) alternariol (AOH) (**5**) [15,16,17], cytosporone C (**6**) [18,19], and the new natural product 2-hydroxyalternariol (**7**).

**Figure 2:**
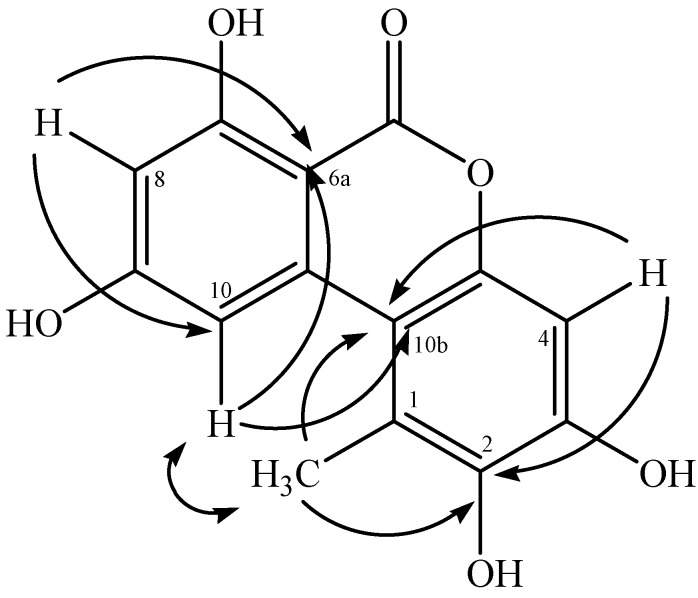
Key HMBC (→) and NOESY (↔) correlations for 2-hydroxy-alternariol (**7**).

Compound **7** was obtained as a white amorphous powder, and its HRMS-ESI data indicated the molecular formula C_14_H_10_O_6_ ([M−H]^−^) from *m/z* 273.0363 (calcd for C_14_H_9_O_6_ 273.0420). The ^1^H-NMR (Table 1) spectrum indicated the presence of two aromatic signals at *δ*_H_ 6.34 (*d*, 1H, *J* = 2.5 Hz, H-8) and at *δ*_H_ 7.26 (*d*, 1H, *J* = 2.5 Hz, H-10). The ^1^H-^1^H COSY spectrum showed correlation between both signals, suggesting a tetrasubstituted aromatic ring system. Additionally, it was observed the proton signal at *δ*_H_ 6.68 (1H, *s*, H-4), suggesting the second aromatic ring system was pentasubstituted. HMBC (Figure 2) spectrum provided the connection of H-8 to C-6a/C-10, suggesting the ring A. The correlation of H-4 and 1-CH_3_ to C-10b/C-2, suggesting the ring C. HMBC correlation of H-10 with C-10b indicated the connection for both aromatic rings. The signals of aromatic ring **A** obtained in **7** was consistent with NMR data reported to AOH [15,16], suggesting its derivative. Thus, compound **7** was identified as 2-hydroxy-alternariol, a new natural product.

**Table 1:** .^1^H (500 MHz) and ^13^C-NMR (125 MHz) spectral data for **7** in DMSO*-d_6_*.

	7	
Position	*δ_H_*,mult. (*J* in Hz)	*δ_C_*
1	-	122.0
2	-	141.7
3	-	146.5
4	6.68 ( *s*)	100.7
5	-	*
6	-	*
6a	-	97.4
7	-	164.4
8	6.34 ( *d*, 2.5 Hz)	100.5
9	-	165.0
10	7.26 ( *d*, 2.5 Hz)	104.1
10a	-	*
10b	-	109.0
1-CH_3_	2.58 ( *s*)	18.8

* not detected.

Compound **7** was previously reported by Pfeiffer *et al.* [20] as a biotransformation compound from AOH originating in the microsomes of rat, human and porcine liver. The mass spectrum of compound **7** was compared with the crude extract obtained from *Phomopsis* sp. by HPLC-MS, which confirmed that **7** is a new natural product and not an artifact. This report is the first to present NMR data for this compound.

**Figure 3:**
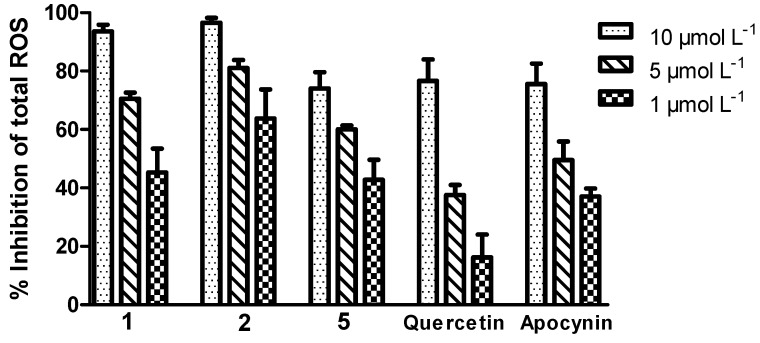
The ability of the tested compounds and standards to inhibit the total ROS produced by stimulated neutrophils.

Compounds **1**, **2** and **5** exhibited a potent inhibitory effect on ROS produced by stimulated neutrophils (Figure 3). Compound **2** exhibited the most significant inhibitory effect (IC_50_ = 0.91 ± 0.26 µmol L^−1^) with an inhibitory concentration exceeding those of apocynin (IC_50_ = 3.90 ± 0.30 µmol L^−1^), which is an efficient inhibitor of the NADPH oxidase complex [21], and quercetin (IC_50_ = 4.86 ± 0.36 µmol L^−1^), a flavonoid with potent antioxidant activity [22]. Compounds **1**, **2** and **5** were able to modulate the neutrophilic ROS generation triggered by the zymosan opsonized stimuli in a concentration-dependent manner.

Of the ROS generated by the neutrophils, HOCl is the most potent oxidant. The product of the myeloperoxidase (MPO)-catalyzed oxidation of chloride in neutrophils, HOCl, plays an important role in the immune system by killing invading bacteria, and its excessive production has been linked to the progression of a variety of diseases including atherosclerosis, rheumatoid arthritis, certain inflammatory cancers, kidney diseases [23,24,25].

Compounds **1**, **2** and **5** were HOCl scavengers, with IC_50_ values of 17.05 ± 0.67, 16.25 ± 0.67 and 16.48 ± 0.79 µmol L^−1^, respectively. Quercetin, an antioxidant used as a reference, exhibited high scavenging capacity (IC_50_ = 3.631 ± 0.29 µmol L^−1^), which can be attributed to the presence of the 3'- and 4'-hydroxyl groups (catechol groups) [22]. Compound **4** showed an IC_50_ exceeding 50 µmol L^−1^; compounds **3**, **6** and **7** were not tested because an insufficient quantity was isolated.

Compounds **1**, **2**, **4** and **5** were tested for their MPO enzymatic activity and as scavengers of the superoxide anion (the first ROS produced through NADPH oxidase activation in neutrophils) but were inactive, showing IC_50_ values exceeding 50 µmol L^−1^. 

Compounds **1**, **2** and **5** did not act by inhibiting the MPO chlorinating activity or by scavenging the superoxide anion, but efficiently inhibited the ROS produced by neutrophils, which indicates that these compounds may act as NADPH oxidase complex inhibitors, thus, reducing the ability of neutrophils to produce HOCl, which could prove beneficial for the prevention of oxidative damage [23].

To investigate whether a possible toxic effect of **1**, **2**, **4** and **5** caused the decrease in neutrophil function, the cytotoxicity of these compounds was evaluated using a trypan blue exclusion assay (Table 2). Compounds **1**, **2**, **4** and **5** were nontoxic to human neutrophils at concentrations below 100 µmol L^−1^ at 30 min and after 60 min. At a concentration of 10 µmol L^−1^ (at which a high inhibition of oxidative neutrophil metabolism has been verified), the viability of the cells exceeded 98%. These results indicate that the inhibitory effects were not mediated through cell death.

**Table 2:** Evaluation of the cytotoxicity of compounds **1**, **2**, **4** and **5** at different times and concentrations.

Compounds	Concentration (µmol L^−1^)	Viable cells (%) *	
		30 min	60 min
Control		99.0 ± 0.60	99.0 ± 1.41
1	100	80.0 ± 2.80	50.0 ± 2.80
1	10	97.0 ± 0.70	97.0 ± 0.70
2	100	85.0 ± 1.40	60.0 ± 2.80
2	10	96.0 ± 0.70	96.0 ± 1.41
4	100	85.0 ± 3.50	50.0 ± 2.80
4	10	95.0 ± 1.41	95.0 ± 0.70
5	100	70.0 ± 2.80	45.0 ± 2.80
5	10	96.0 ± 0.70	96.0 ± 0.70

* The data are expressed as the means ± the standard deviation; *n* = 3. Each compound was assayed in triplicate.

A classic assay for DPPH^●^ radical scavenger capacity was performed, but all compounds tested were inactive, with IC_50_ values exceeding 50 µmol L^−1^.

Cytochalasin H (**2**) exhibited activity against *C. Cladosporioides* and *C. sphaerosphermum* (the minimum quantities required to inhibit fungal growth were 10.0 and 25.0 µg, respectively) with nystatin used as the reference (the minimum quantity required to inhibit fungal growth was 1.0 µg). Cytochalasin H (**2**) exhibited acetylcholinesterase activity (the minimum quantity required to inhibit acetylcholinesterase was 25.0 µg), and the physostigmine was used as reference (the minimum quantity required to inhibit fungal growth was 1.0 µg). The other compounds were inactive in these assays.

The cytochalasins are a group of secondary fungal metabolites structurally complex. They have been found in several fungal genus, such as *Ascochyta* sp., *Aspergillus* sp., *Phomopsis* sp., *Turbercularia* sp., *Xylaria* sp., among others. These substances have a wide range of biological activities including inhibition of HIV-1 protease 2, as well as antibiotic and cytotoxic activities [26,27].

The chemical and biological study of the CH_3_CN fraction from *Phomopsis* sp. led to the isolation of mycotoxins with potent bioactivity. AOH (**5**) and AME (**4**) are mycotoxins commonly found in foods contaminated with *Alternaria alternate* [4]. A previous study demonstrated that consuming food contaminated with these toxins can increase the incidence of esophageal cancer, and there have been several reports concerning the mutagenicity and genotoxicity of AOH and AME [5,20,28].

## 3. Experimental Section

### 3.1. General Experimental Procedures

The ^1^H-NMR (500 MHz), ^13^C-NMR (125 MHz), *g*HMBC, *g*HMQC and *g*COSY experiments were recorded on a Varian DRX-500 spectrometer using the non-deuterated residual signal as the reference. The mass spectra were measured on a LC–MS Q-TOF Micromass spectrometer in the ESI mode with MeOH as the eluent (cone voltage = 25 V). The TLC was performed using Merck silica gel 60 (230 mesh). The spots on the TLC plates were observed under UV light by spraying with anisaldehyde-H_2_SO_4_ followed by heating to 100 °C. Preparative HPLC was performed on a Shimadzu system coupled with a UV SPD detector using a Phenyl preparative column (250 mm × 2.0 mm). Analytical HPLC was performed on a Shimadzu system coupled with a UV SPD detector system using a phenyl column (25.0 cm × 3.0 cm). Column chromatography (CC) was performed using silica gel (0.060–0.200 mm; Acros Organics, Fair Lawn, NJ, USA). Optical rotations were measured on a PerkinElmer polarimeter with a sodium lamp operating at 589 nm at 25 °C. IR spectra were obtained with a PerkinElmer FTIR-1600 spectrophotometer using KBr pellets.

Taurine, calcium chloride, magnesium chloride, glucose, catalase (EC 1.11.1.6), dimethyl sulfoxide (DMSO), luminol (5-amino-2,3-dihydro-1,4-phthalazinedione), 2,2-diphenyl-1-picrylhydrazyl (DPPH^●^), 5,5'-tetramethylbenzidine (TMB), zymosan, Histopaque^®^-1077 and Histopaque^®^-1119 were purchased from Sigma–Aldrich Chemical Co. (St. Louis, MO, USA). 2-(4-Iodophenyl)-3-(4-nitrophenyl)-5-(2,4-disulfophenyl)-2H-tetrazolium monosodium salt (WST-1) was purchased from Santa Cruz (Santa Cruz, CA, USA). MPO (EC 1.11.1.7) was purchased from Planta Natural Products (Vienna, Austria), and its concentration was determined from its absorption at 430 nm (ɛ = 89,000 mol^−1^ cm^−1^ per heme) [29]. Hydrogen peroxide was prepared by diluting a 30% stock solution purchased from Peroxidos do Brazil (Sao Paulo, SP, Brazil), and its concentration was calculated using its absorption at 240 nm (ɛ = 43.6 mol^−1^ cm^−1^) [30]. Hypochlorous acid was prepared by diluting a concentrated commercial bleach solution, and its concentration was calculated from its absorption at 292 nm (ɛ = 350 mol^−1^ cm^−1^) [29]. All solutions were prepared with water purified in a Milli-Q system (Millipore, Bedford, MA, USA). All reagents used to prepare the buffers were of analytical grade.

### 3.2. Plant Material

Authenticated *Senna spectabilis* (Fabaceae) was collected next to the Chemistry Institute—UNESP, Araraquara, São Paulo, Brazil, in February, 2007. A voucher specimen was deposited at the Herbarium of the Institute of Botany of São Paulo, Brazil (voucher No. SP 384109).

### 3.3. Isolation and Identification of the Endophytes

The endophytic fungus Cs-c2 was isolated from healthy adult leaves of *Senna spectabilis* according to reported methods [31]. The fungus was identified by morphology analysis by the André Tosello Foundation, Campinas—SP and deposited in the NuBBE collection under the number Cs-c2 for *Phomopsis* sp.

### 3.4. Fermentation, Extraction and Isolation

The endophytic fungus strain *Phomopsis* sp. was cultivated in eight Erlenmeyer flasks (500 mL), each containing 90 g of corn meal and 75 mL of H_2_O. The medium was autoclaved three times (on three consecutive days) at 121 °C for 20 min. After sterilization, the medium was inoculated with the endophyte and incubated while stationary at 26 °C for 21 days. At the end of the incubation period, the cultures were combined, ground and extracted with CH_3_OH (7 × 200 mL). The solvent was evaporated to yield a crude CH_3_OH extract (21.0 g). The CH_3_OH extract was dissolved in CH_3_CN (500 mL) and defatted with hexane via liquid partitioning. The CH_3_CN fraction was then evaporated to yield 2.90 g.

The CH_3_CN fraction was fractionated via column chromatography on a silica gel column (2.5 cm × 27.0 cm) and eluted with a CHCl_3_/CH_3_OH gradient (1%–100% MeOH) to yield 31 sub-fractions (Psp01-31), which were retained based on their similarity with the TLC profiles. The sub-fraction Psp18-20 (360.5 mg) was submitted to preparative HPLC separation using H_2_O/CH_3_OH (60:40 v/v until 0:100% over 40 min, 10 mL min^−1^, λ_max_ = 235 nm) as the eluent to yield compounds 1 (15.1 mg, R_T_ = 24.8 min), 2 (103.5 mg, *R*_T_ = 28.1 min), **3** (1.2 mg, *R*_T_ = 21.5 min) and **4** (40.0 mg, *R*_T_ = 31.5 min). CHCl_3_ (15 mL) was added to the sub-fractions Psp25 and Psp30, the substances **5** + **6** (9.2 mg) and **7** (4.2 mg), respectively, were obtained after stirring and filtration of the mixture. Compounds **5** + **6** were purified via preparative HPLC using H_2_O/CH_3_OH (45:55 until 20:80 over 20 min at a rate of 10 mL min^−1^, λ_max_ = 235 nm) as the eluent to yield **5** (6.0 mg) and **6** (1.2 mg).

### 3.5. Biological Activity

#### 3.5.1. The Reactive Oxygen Species (ROS) Inhibitory Activity was Measured Using a Cellular Assay

The total ROS produced by the stimulated neutrophils was measured using luminol-enhanced chemiluminescence (LumCL) assays. The human neutrophils were isolated using blood samples obtained from healthy volunteers. The experiments were performed in accordance with the regulations of the Research Ethics Committee (29/2011, Faculty of Pharmaceutical Sciences, UNESP, São Paulo, Brazil). The neutrophils were isolated and the LumCL assay was performed according to previously reported methods [32,33].

#### 3.5.2. Myeloperoxidase (MPO) Inhibitory Activity

The chlorination activity of the MPO was based on the reaction of HOCl with taurine to produce taurine chloramine, which oxidizes TMB [34]. The resulting oxidation product was detected spectrophotometrically at 655 nm using a microplate reader (Synergy 2 Multi-Mode, BioTek, Winooski, VT, USA), according to the procedure described by Zeraik *et al.* [35].

#### 3.5.3. Antioxidant Capacity

The scavenging capacity of compounds **1**, **2**, **4** and **5** was evaluated using the potent oxidant HOCl (a compound generated by MPO), O_2_^●−^ (produced during a respiratory burst by NADPH oxidase) and DPPH^●^ (the classic method for measuring antioxidant capacity). The abilities of **1**, **2**, **4** and **5** to scavenge HOCl were determined by measuring the oxidation of TMB by taurine chloramine at 655 nm using a microplate reader (Synergy 2 Multi-Mode, BioTek) according to the procedure described by Ximenes *et al.* [36]. The superoxide anion radical generated from the xanthine/xanthine oxidase system (X/XO) was studied by reducing the tetrazolium salt, WST-1, to produce a soluble formazan superoxide according to the modified method proposed by Tan and Berridge [37]. The DPPH^●^ method was also performed to verify the antioxidant capacity of **1**, **2**, **4** and **5**. The method developed by Brand-Williams *et al.* [38] was followed with certain modifications [39]. Quercetin was used as a positive control under different concentrations in DMSO.

#### 3.5.4. Cytotoxic Activity

The cytotoxic effect of compounds **1**, **2**, **4** and **5** on human neutrophils was studied using the trypan blue exclusion assay according to the method by Kitawaga *et al.* [33].

#### 3.5.5. Antifungal Activity

*Cladosporium cladosporioides* (Fresen) de Vries CCIBt 140 and *C. sphaerospermum* (Penzig) CCIBt 491 were used in the antifungal assay. Compounds **1**, **2**, **4**, **5** and **7** were applied to pre-coated Si-gel TLC plates using a solution (10 mL) containing 100.0, 50.0, 25.0, 10.0, 5.00 and 1.00 µg samples of the pure compounds. After eluting with CHCl_3_/CH_3_OH (9:1), the plates were sprayed with the fungal suspension [40]. Nystatin was used as the positive control at 1.0 µg.

#### 3.5.6. Acetylcholinesterase Inhibitory Activity

Compounds **1**, **2**, **4**, **5** and **7** were investigated (elution of the TLC with the CHCl_3_/CH_3_OH (9:1)) according to reported methods [41]. Galantamine was employed as the positive control at 1.0 µg.

## 4. Conclusions

The production of cytochalasins with known potent biological activity [10,26], such as cytochalasin H (**2**), with demonstrated activity against pathogenic fungi and the production of mycotoxins reinforce the hypothesis that symbiosis between the endophyte and the host plant can produce substances that show antifungal activity against possible phytopathogenic fungi or are harmful to predators of the plant. Additionally, cytochalasins J (**1**) and H (**2**) and alternariol were found to have potent inhibitory effects on human neutrophils by acting as potential inhibitors of NADPH oxidase and may be promising targets for the development of anti-inflammatory agents. This study of the fungus *Phomopsis* sp. revealed that the endophyte produces bioactive metabolites, thus justifying further chemical study of this class of microorganisms. 

## Supplementary Materials

Supplementary materials can be accessed at: http://www.mdpi.com/1420-3049/19/5/6597/s1.
