# Supplementary material for: Bioactive Secondary Metabolites from Phomopsis sp., an Endophytic Fungus from Senna spectabilis
Source: Molecules. 2014 May 22;19(5):6597–608. doi: 10.3390/molecules19056597 (PMC6271730; doi:10.3390/molecules19056597)

Supplementary Material for
Bioactive Secondary Metabolites from *Phomopsis* sp., an Endophytic Fungus from *Senna spectabilis*

NMR spectra of compound **7**. **S1–S3**

MS spectrum of compound **7**. **S4**

Figure S1. ^{1}H-NMR spectrum (500MHz, DMSO-d_6) of **7.**

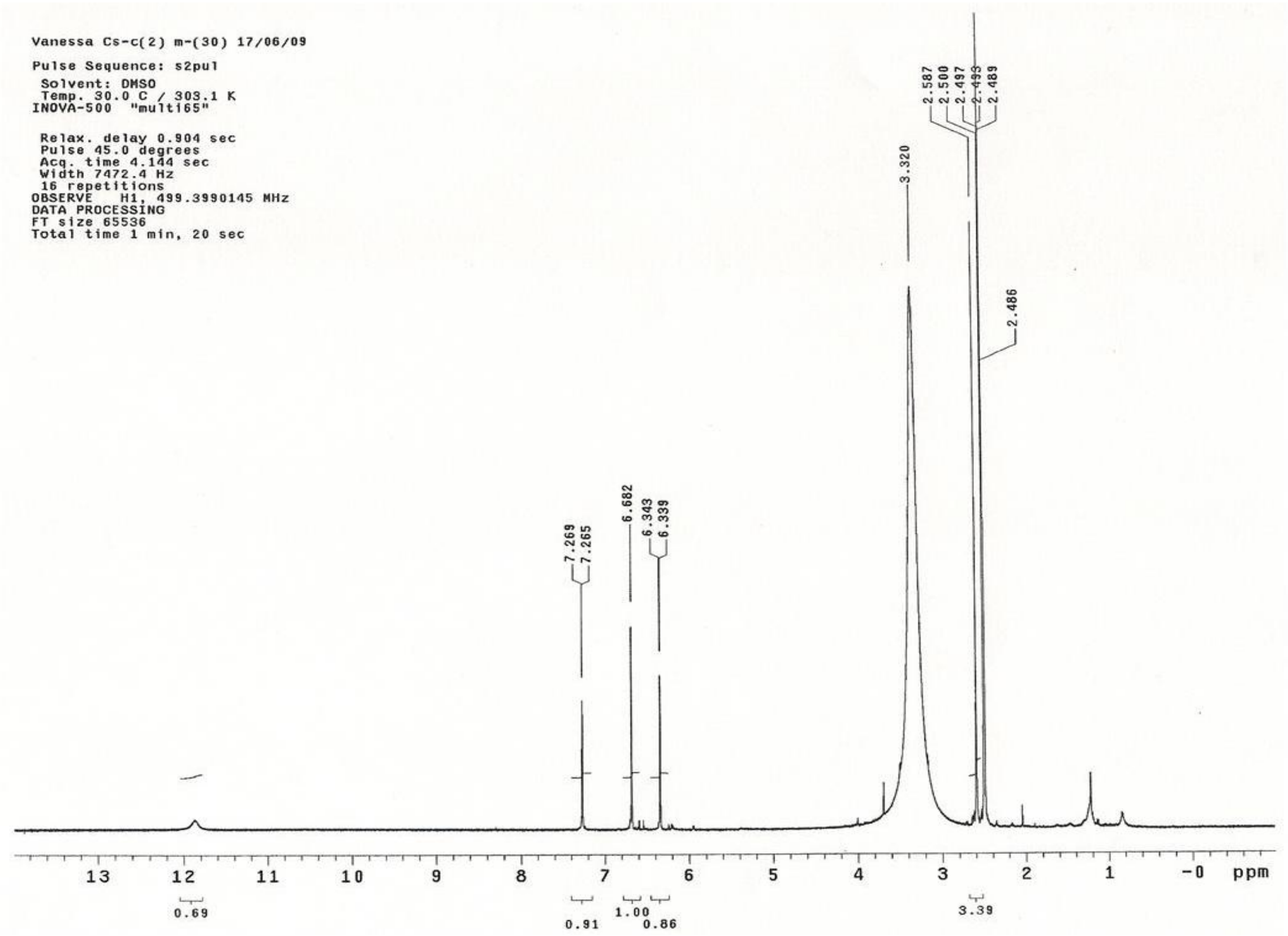

Figure S2. *g*HMBC spectrum (DMSO-d_6) of **7**.

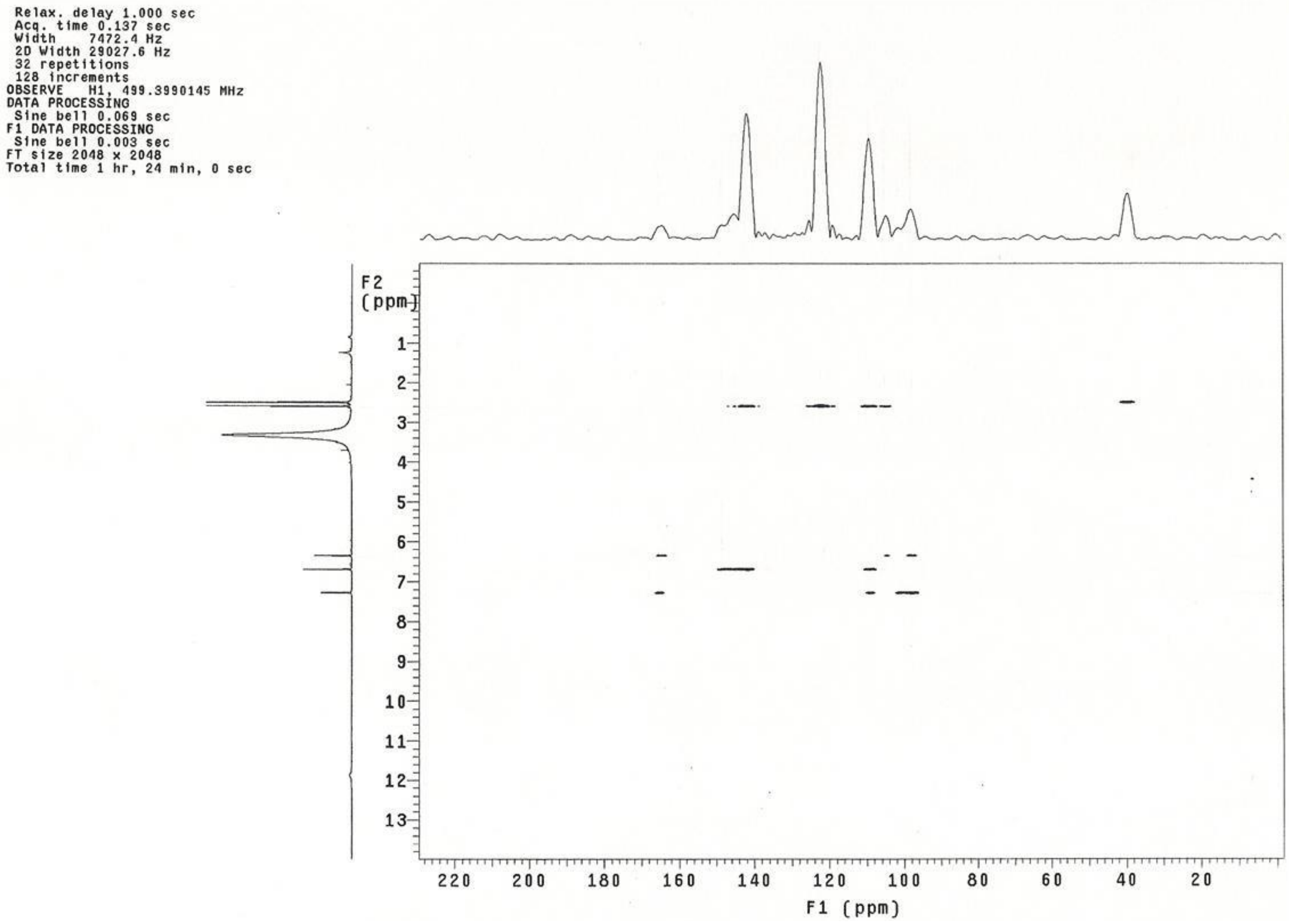

Figure S3. *g*HMQC spectrum (DMSO-d_6) of **7**.

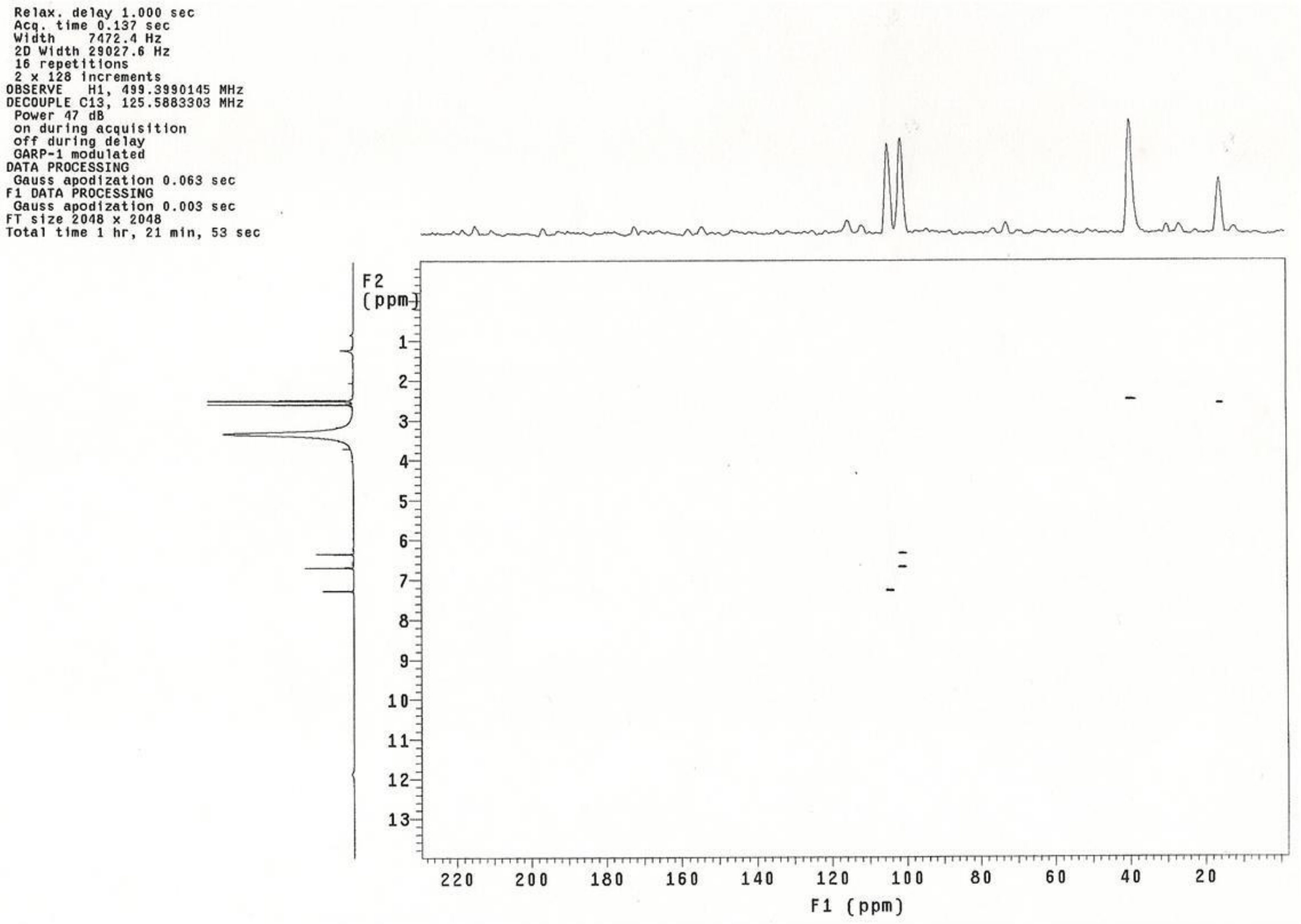

Figure S4. Mass espectrum of **7**.

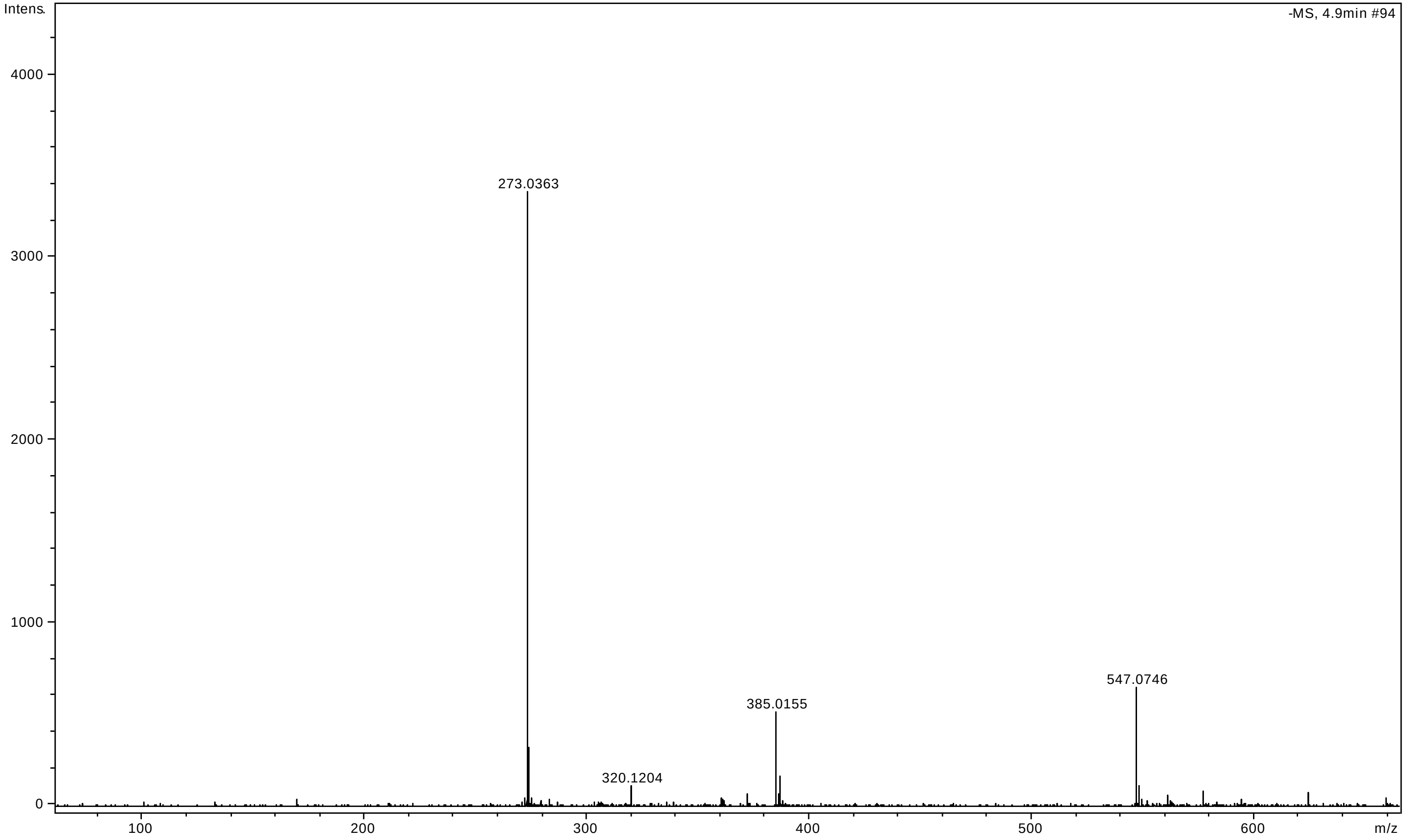

Supplement: Supplementary file 1 [file molecules-19-06597-s001.pdf]
